# Effect of urban design on microclimate and thermal comfort outdoors in warm-humid Dar es Salaam, Tanzania

**DOI:** 10.1007/s00484-017-1380-7

**Published:** 2017-06-14

**Authors:** Moohammed Wasim Yahia, Erik Johansson, Sofia Thorsson, Fredrik Lindberg, Maria Isabel Rasmussen

**Affiliations:** 1 Housing Development & Management, Department of Architecture and Built Environment, Lund University, Lund, Sweden; 2 Department of Earth Sciences, University of Gothenburg, Gothenburg, Sweden

## Abstract

Due to the complexity of built environment, urban design patterns considerably affect the microclimate and outdoor thermal comfort in a given urban morphology. Variables such as building heights and orientations, spaces between buildings, plot coverage alter solar access, wind speed and direction at street level. To improve microclimate and comfort conditions urban design elements including vegetation and shading devices can be used. In warm-humid Dar es Salaam, the climate consideration in urban design has received little attention although the urban planning authorities try to develop the quality of planning and design. The main aim of this study is to investigate the relationship between urban design, urban microclimate, and outdoor comfort in four built-up areas with different morphologies including low-, medium-, and high-rise buildings. The study mainly concentrates on the warm season but a comparison with the thermal comfort conditions in the cool season is made for one of the areas. Air temperature, wind speed, mean radiant temperature (MRT), and the physiologically equivalent temperature (PET) are simulated using ENVI-met to highlight the strengths and weaknesses of the existing urban design. An analysis of the distribution of MRT in the areas showed that the area with low-rise buildings had the highest frequency of high MRTs and the lowest frequency of low MRTs. The study illustrates that areas with low-rise buildings lead to more stressful urban spaces than areas with high-rise buildings. It is also shown that the use of dense trees helps to enhance the thermal comfort conditions, i.e., reduce heat stress. However, vegetation might negatively affect the wind ventilation. Nevertheless, a sensitivity analysis shows that the provision of shade is a more efficient way to reduce PET than increases in wind speed, given the prevailing sun and wind conditions in Dar es Salaam. To mitigate heat stress in Dar es Salaam, a set of recommendations and guidelines on how to develop the existing situation from microclimate and thermal comfort perspectives is outlined. Such recommendations will help architects and urban designers to increase the quality of the outdoor environment and demonstrate the need to create better urban spaces in harmony with microclimate and thermal comfort.

## Introduction

The projected higher air temperature and more frequent periods of extensive heat due to global warming is expected to become a serious problem in warm climates (Ndetto and Matzarakis 2015).

There have been several studies on thermal conditions of cities in warm-humid climates in later years (e.g., Johansson and Emmanuel 2006; Lin 2009; Johansson and Yahia 2011; Ng and Cheng 2012; Yang et al. 2013; Ndetto and Matzarakis 2013, 2015, 2016). These studies have shown that thermal conditions are stressful for the urban dwellers and that increased air temperatures will not only lead to increased heat stress but also to increased energy use for air-conditioning and consequently increased emissions of greenhouse gases.

Urban design has a significant impact on microclimate and outdoor thermal comfort. Several studies in warm-humid climates have concluded that ventilation and shade are crucial to improve thermal comfort (Chen et al. 2001; Ng 2009; Hong et al. 2011, 2015). However, often the thermal conditions are worsened as a consequence of poor urban design including lack of shade and poor ventilation (Hong et al. 2015). This in turn leads to increased occurrence of heat stress and heat-related diseases as well as diminished performance of both mental and physical tasks (Better Health Channel 2012). High-rise buildings reduce the radiant heat load, i.e., mean radiant temperature (MRT) at pedestrian level compared to low-rise (Emmanuel et al. 2007; Johansson et al. 2013). However at solar noon, high-rise buildings alone cannot create shade due to the high solar elevations. This implies that additional measures, such as the use of vegetation or shading devices, must be taken to reduce the radiant heat load at lower latitudes at noon. In warm and humid regions, it has been found that on average about 80% of the total cooling effect of the sites is contributed by tree shading (Yoshida et al. 2000; Shashua-Bar and Hoffman 2000).

To allow for good ventilation, the city form cannot be too dense (Ng 2009). Barriers of buildings as well as vegetation (especially trees) decrease wind speed at street level (Feng et al. 2009) and might block dominant wind directions or local wind systems, such as for example the sea breeze, which help to bring cooler air from the sea into the land in the afternoon (Ribeiro et al. 2016). Although vegetation in general has a positive effect on outdoor thermal comfort (Shashua-Bar et al. 2011), blocking the air movement at the pedestrian level by additional plantation should be avoided in warm climates (Emmanuel and Johansson 2006, Ng 2009). To some extent, there is thus a conflict between good ventilation and sufficient shade; a compromise has to be found.

Although several studies on outdoor thermal comfort in warm-humid cities have been carried out lately, there are still few studies from African cities including Tanzania. However, already in the 1970s, Nieuwolt (1973) argued that the effect of sea breeze could improve the thermal comfort in Dar es Salaam. Recent studies conducted in Dar es Salaam have shown that the MRT may exceed 45 °C at noon time from late November to early March. In addition, the physiologically equivalent temperature (PET) may reach values of up to 41 °C, i.e., extreme heat stress, during the afternoons from November to April in east-west oriented streets (Ndetto and Matzarakis 2013, 2015). The same authors have estimated the thermal comfort range in Dar es Salaam to be 23–31 °C of PET based on micrometeorological measurements and questionnaires (Ndetto and Matzarakis 2016). However, the wind speed used in the calculations was taken from the airport and not at the sites of investigation which might have affected the mean radiant temperature and consequently PET.

The aim of this study is to evaluate the effect of different urban morphologies on the microclimate, especially wind speed, solar exposure, and shade in the city of Dar es Salaam. In addition, the temporal and spatial variation in human comfort outdoors, expressed by PET is investigated. The study compares four different neighborhoods with distinctly different urban design, i.e., different street patterns, built area coverage, building heights, and amount of vegetation.

## Material and methods

### The city of Dar es Salaam

**Fig. 1:**
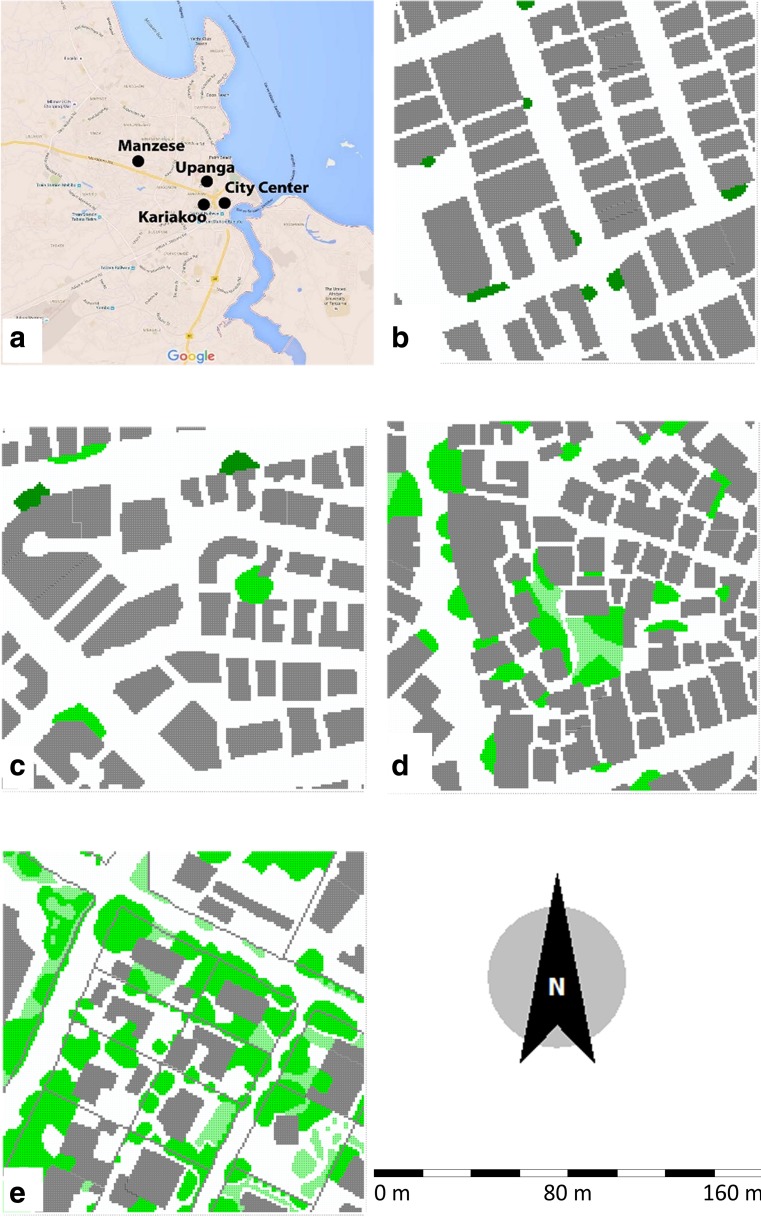
**a** Map of Dar es Salaam showing the four studied areas as well as footprints of the studied areas as ENVI-met input files, where **b** Kariakoo, **c** City Center, **d** Manzese, and **e** Upanga

Dar es Salaam is located on the eastern coast of the country along the Indian Ocean at latitude 6° 48′ S and longitude 39° 17′ E (see Fig. 1). The city, which is situated on a relatively flat coastal plain, is the largest city in Tanzania with a population of over four millions in the metropolitan area. Due to rapid urbanization the city nearly doubled its population in the last two decades (Ndetto and Matzarakis 2015). The annual growth of 4.4% is one of the highest in the world (Ndetto and Matzarakis 2013) and as a consequence, an estimated 70% of the city’s population are expected to live in informal (unplanned) neighborhoods (Rasmussen 2013). Dar es Salaam is characterized by a radial structure with settlements around the City Center. Informal settlements of middle and low-income families have emerged in between the main roads in a pattern of compact, low-rise residential areas, contributing to inefficient use of land (Rasmussen 2013; Lupala 2002).

### The climate in Dar es Salaam

The climate of Dar es Salaam is warm-humid, and affected by the monsoons. Between May and October, the monsoon blows from southeast whereas between December and March, the city is affected by the northeast monsoon (Nieuwolt 1973). The annual mean air temperature is about 26 °C with slight seasonal changes due to the proximity to the equator. The period from May to September is the coolest (the average air temperature is about 24 °C) whereas the warmest period is between December and March with an average of about 28 °C. Most rainfall occurs between March and May (120–260 mm/month on average) whereas the period June to October is dry (about 50 mm/month as average). Generally, the monthly relative humidity in Dar es Salaam varies between 77 and 86% over the year. The average value of vapor pressure is considerably higher during the warm and wet season (29 hPa in February) than in the cool and dry season (23 hPa in July). In this study, the months February and July were selected as representative of the warmest and coolest periods, respectively.

### Selection of the study areas

**Fig. 2:**
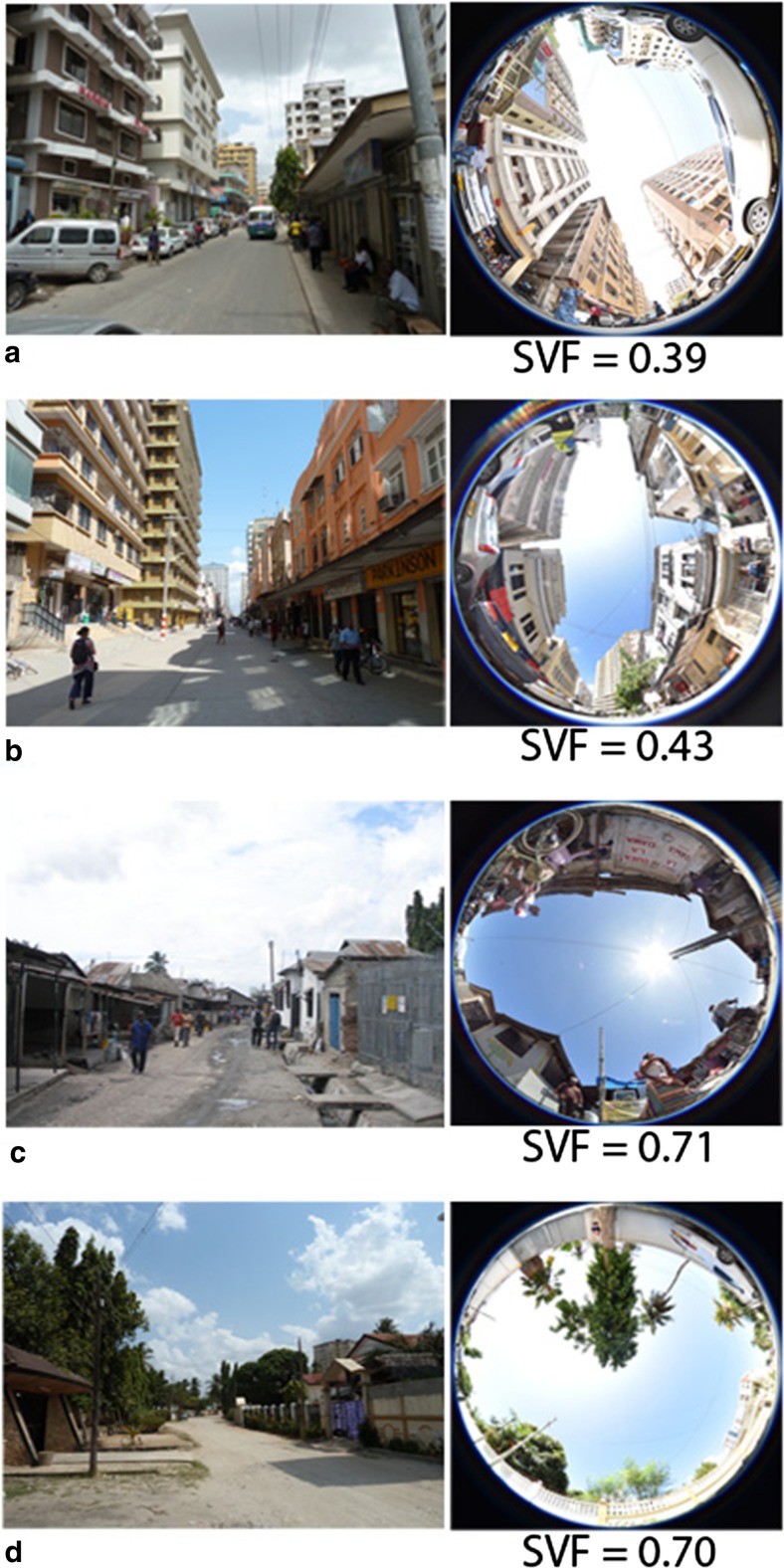
Street photographs and sky-view photos from typical streets in each of the four studies areas where **a** Kariakoo, **b** City Centre, **c** Manzese, and **d** Upanga

This paper concentrates on the neighborhoods Kariakoo, City Center, Manzese, and Upanga which have distinctly different characteristics as regards urban morphology and building types (see Figs. 1 and 2).

The neighborhood Kariakoo has an orthogonal street pattern with a large central market place. Buildings were originally one or two-story so-called Swahili houses, following local traditional patterns. In the middle of the twentieth century, medium-rise buildings of 3 to 4 stories started to be built and replaced partly the Swahili houses. High-rise buildings have been introduced in recent years. The area is practically devoid of vegetation (MLHHSD 2002; Lupala 2002). The City Center of Dar es Salaam faces the Indian Ocean. Its southwestern part, in which the study area is located, is mainly characterized by three to four-story buildings and vegetation in the streets is scarce (Lupala 2002). The rapid urbanization and recent years’ economic growth has resulted in extensive urban development in the City Center, leading to the transformation of low- and medium-rise buildings into high-rise apartment complexes. The informal neighborhood Manzese, which is located more inland, represents one of the most common types of urban morphology in Dar es Salaam. The study area mainly comprises simple single- or two-story houses that follow the traditional Swahili house with a compact layout, narrow labyrinthine and unpaved streets in an organic urban fabric. The neighborhood of Upanga, which was planned as a European garden-city type with winding long streets and villas surrounded by greenery, consists of mainly two to three-story buildings, including both apartment buildings and individual houses; recently areas of high-rise buildings have started to be built. The area is characterized by tree-lined streets and trees are also very commonly found in front- and backyards. Figure 2 shows street photographs and sky-view photos from the four studies areas.

### Simulation procedure

The impact of different urban morphologies on the microclimate thermal comfort was simulated by using ENVI-met 3.1 (Bruse 2015). Most simulations were carried out for 28th February (typical warm day) which was chosen to represent the period February–March which is the warmest according to the local meteorological data for Dar es Salaam. Complementary simulations were carried out in the area Upanga for the cool period represented by 15th July (typical cool day). The simulated period lasted from 5:00 local time (LT) in the morning until 16:00 in the afternoon to include the maximum air temperature and MRT, which occur in the afternoon.

Thermal comfort, sky view factor (SVF), built area coverage and floor area ratio (FAR) in four different urban morphologies were examined. The building foot prints were directly imported using metadata from a satellite image (raster data). The building heights were measured on site using a Nikon® Laser 550 Rangefinder. The four simulated areas have the same model size 160 m × 160 m (the model grid resolution was 1 m in directions dx and dy). The building heights varied from 3 to 55 m and therefore, the model grid resolution for the dz was set as 4 m. The simulated areas are shown in Fig. 1.

### Model calibration

The model was calibrated with on-site long-term measurements of air temperature (*T*_a_), relative humidity (RH), wind speed (*W*_s_) and direction (*W*_d_) at the National Museum in Dar es Salaam as well as properties of ground surface materials. The general input data as a result of the calibration are shown in Table 1. Since ENVI-met underestimates the diurnal *T*_a_ fluctuations (Ali-Toudert and Mayer 2006; Yahia and Johansson 2014), the initial *T*_a_ was overestimated at the start of the simulations to reach the measured values at the maximum *T*_a_ which occurs in the afternoon (the time of interest).

**Table 1:** The basic input configuration data used in the ENVI-met simulations for February and July. The values used are a result of the calibration process

Category	Configuration data	February	July
Time	Start of simulation (h)	5:00	5:00
Meteorology	Wind speed at 10 m above ground level [ms^−1^]	3.8	3.4
	Wind direction [°]	45	150
	Initial temperature of the atmosphere [K]	302	299
	Solar adjustment factor^a^	0.98	0.96
	Specific humidity [gm^−3^]	15	15
	Relative humidity at 2 m [%]	70	62
	Fraction of low clouds (in octas)	3	2
	Fraction of medium clouds (in octas)	2	2
	Fraction of high clouds (in octas)	0	1
Soil	Initial temperature upper layer (0–20 cm) [K]	303	300
	Initial temperature middle layer (20–50 cm) [K]	301	298
	Initial temperature deep layer (below 50 cm) [K]	299	296
Buildings	Albedo of walls for buildings	0.3	0.3
	Albedo of roofs for buildings	0.3	0.3

^a^The solar radiation, which is calculated as a function of the latitude, was slightly over-estimated by ENVI-met for Dar es Salaam conditions in February and July. An adjustment factor was applied to reach maximum global radiation values of 718 and 670 Wm^−2^ for February and July, respectively

### Assessment of microclimate and thermal comfort outdoors

In this study, MRT and PET (Höppe 1999) were calculated. The reason to choose the index PET is that it has been widely used in different climate types including warm humid (Ndetto and Matzarakis 2015, 2016; Johansson et al. 2016). Additionally, PET is expressed in °C which makes it easy to understand by architects and planners.

### The role of vegetation

The impact of vegetation was studied by conducting simulations in Upanga (with and without vegetation).

Trees and grass were found to be the main vegetation types in the studied areas. In this investigation, the green coverage was calculated as the percentage of the green area compared to the total area studied. The green coverage in the areas Kariakoo and City Center is very low (1.2 and 1.6%, respectively), whereas the vegetation in Manzese covers about 10.2% of the total area. In the area Upanga, it is noted that as much as 33.8% (about 1/3) of the total area is covered with vegetation.

The simulated trees in ENVI-met were designed based on observations and two different tree densities—expressed as leaf area index (LAI) and leaf area density (LAD) (Meir et al. 2000)—were applied. Dense trees with 10 m height (LAI = 4.73) and very dense trees with 15 m height (LAI = 9.35) were designed for simulating the vegetation. In addition, grass with 0.1 m height (LAI = 0.03) was also used.

## Results

### Microclimate variations

Table 2 shows that the *T*_a_ mainly varies 4° between 10:00 and 16:00. However, in Manzese, the variation is about 5. This can be explained by the fact that due to the low building heights Manzese receives much more short wave radiation which warms up the air at spaces between buildings more than in other areas. The variation of the RH in the four areas is between 55 and 83% where the highest value occurs at 10:00 and the lowest at 16:00. The specific humidity (*q*) varies between 18 g^−3^ (at 12:00) and about 21 g^−3^ (at 16:00). MRTs temporal variation is about 6–7°, and the highest values occur in Manzese at 14:00 (53 °C) due to the large amount of radiation allowed by the low building heights. In general, the highest MRTs in the four areas are found at 14:00. At this time, the radiation fluxes reach their maximum values when the sun angle is about 70° in February. It is also noted that the maximum *T*_a_ in all areas are recorded at 16:00. Although the *T*_a_ increase between 14:00 and 16:00 in all areas is not decisive (less than 1 °C), it reflects that the net radiation continues to warm up the air even after 14:00.

**Table 2:** Temporal variations (average values at the pedestrian height, 2 m) of air temperature (*T*_a_), relative humidity (RH), specific humidity (*q*), wind speed (*W*_s_), and mean radiant temperature (MRT) in the four studied areas, i.e., City Center, Kariakoo, Manseze, and Upanga in February

Area	Time (h)	T_a_ (°C)	RH (%)	q (gm^−3^)	W_s_ (ms^−1^)	MRT (°C)
Kariakoo	10:00	28.4	80	19.2	0.28	41.2
	12:00	30.5	76	20.6	0.27	46.9
	14:00	31.9	66	19.4	0.28	48.7
	16:00	32.6	58	17.7	0.3	43.5
City Center	10:00	28.6	79	19.2	0.59	44.1
	12:00	30.6	74	20.2	0.58	48.6
	14:00	32.2	63	18.8	0.6	50.2
	16:00	32.8	57	17.6	0.62	45
Manzese	10:00	28.6	81	19.7	0.74	45.6
	12:00	30.6	75	20.5	0.71	49.1
	14:00	32.7	61	18.7	0.73	53
	16:00	33.4	55	17.5	0.76	49.6
Upanga	10:00	28.3	83	19.8	0.62	43.4
	12:00	30.2	78	20.8	0.58	47.8
	14:00	31.7	67	19.4	0.59	50
	16:00	32.5	59	17.9	0.61	45.2

**Fig. 3:**
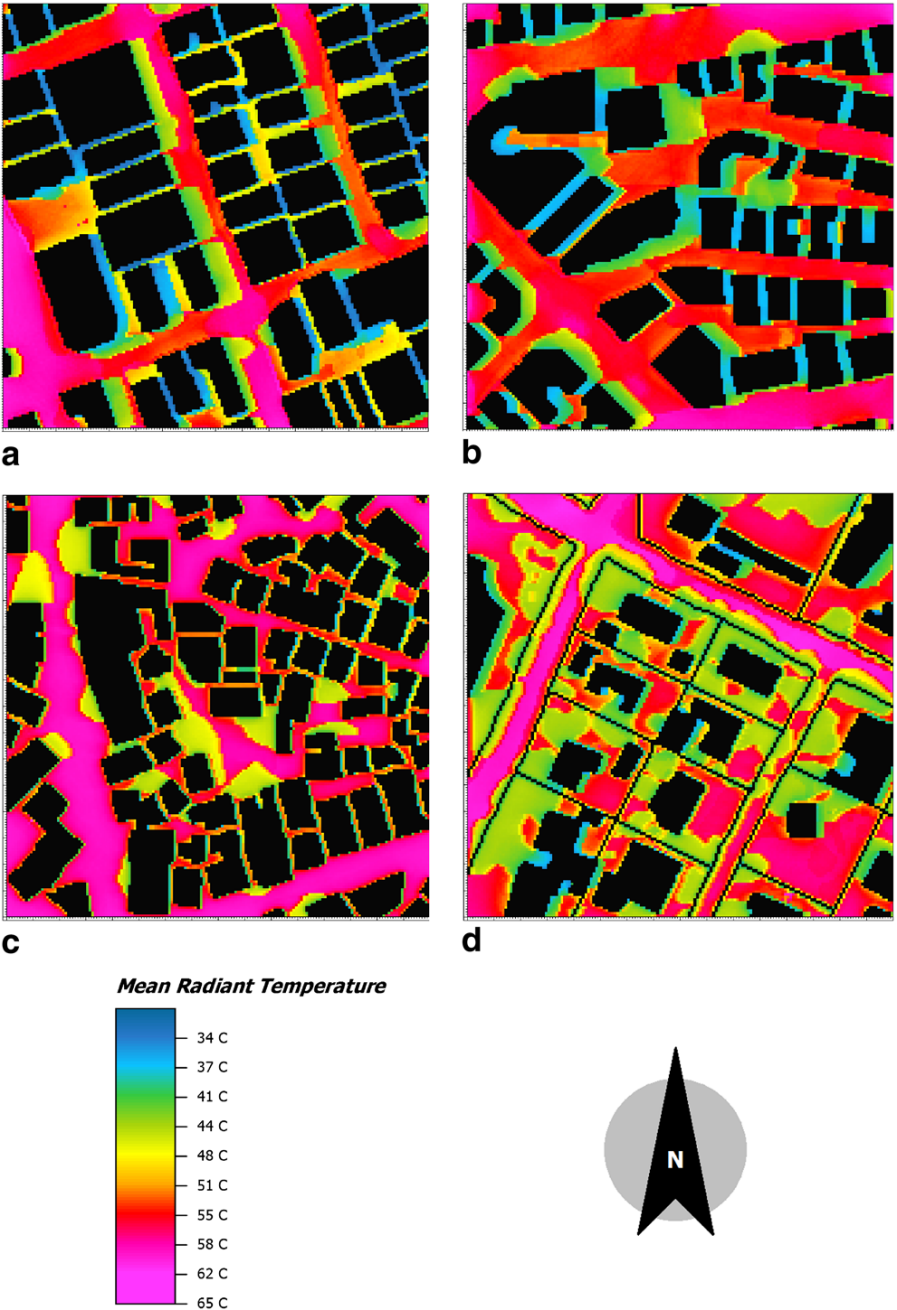
Spatial variation of the mean radiant temperature (MRT) at pedestrian height (2 m) for the areas simulated in February at 14:00 where **a** Kariakoo, **b** City Center, **c** Manzese and **d** Upanga

Figure 3 illustrates a great variation of the radiant heat load at pedestrian height (2 m) within short distances (the minimum and maximum MRTs are 36 and 62 °C, respectively in all areas). In Kariakoo and the City Center, MRT values at the spaces between buildings tend to be lower than at the streets. This is due to the compact urban morphologies with high buildings that provide shading and prevent solar radiation to reach the ground (MRT values are between 34 and 50 °C). On the other hand, in Manzese and Upanga, the spaces between buildings receive more solar radiation compared to Kariakoo and the City Center due to the low building heights. However, Upanga has detached buildings and plenty of vegetation which reduces the MRT values at the spaces between buildings, and thus the MRT values tend to be lower than in Manzese although Manzese has more compact morphology and similar building heights (3–6 m). These results agree with other studies in tropical climates such as Emmanuel et al. (2007) and Johansson et al. (2013).

**Fig. 4:**
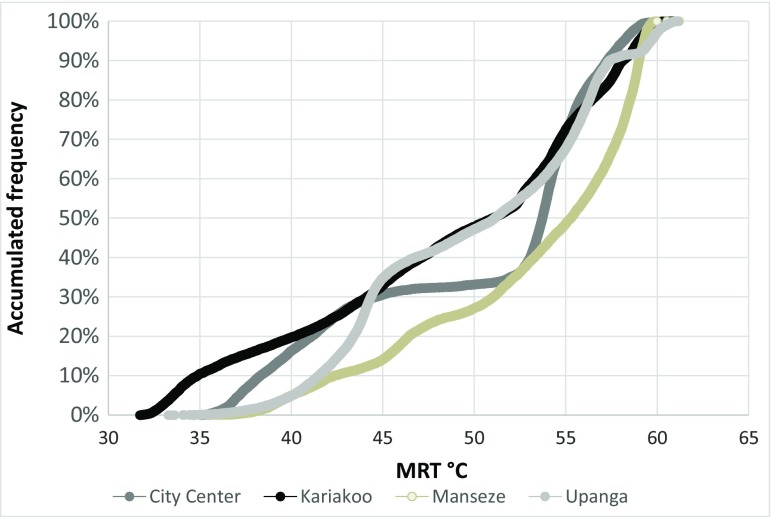
Accumulated frequency of the mean radiant temperature (MRT) in February from 10:00 to 16:00 in the four studied areas, i.e., City Center, Kariakoo, Manzese, and Upanga

Figure 4 shows the accumulated frequency of MRT in February from 10:00 to 16:00 in the four areas. The figure reveals that Manzese has a low frequency of low MRTs whereas it has high frequency of high MRTs. Kariakoo shows an even distribution of MRTs in the whole range between 32 and 59 °C. In Upanga, the MRTs are evenly distributed especially between 45 and 57 °C but there is a very low frequency of low MRTs (between 33 and 40 °C). In the City Center, MRTs are evenly distributed between 37 and 45 °C as well as between 53 and 58 °C. However, there are few MRTs between 46 and 52 °C. This implies that Manzese is very exposed to solar radiation whereas Kariakoo and the City Center have more areas with shade.

**Fig. 5:**
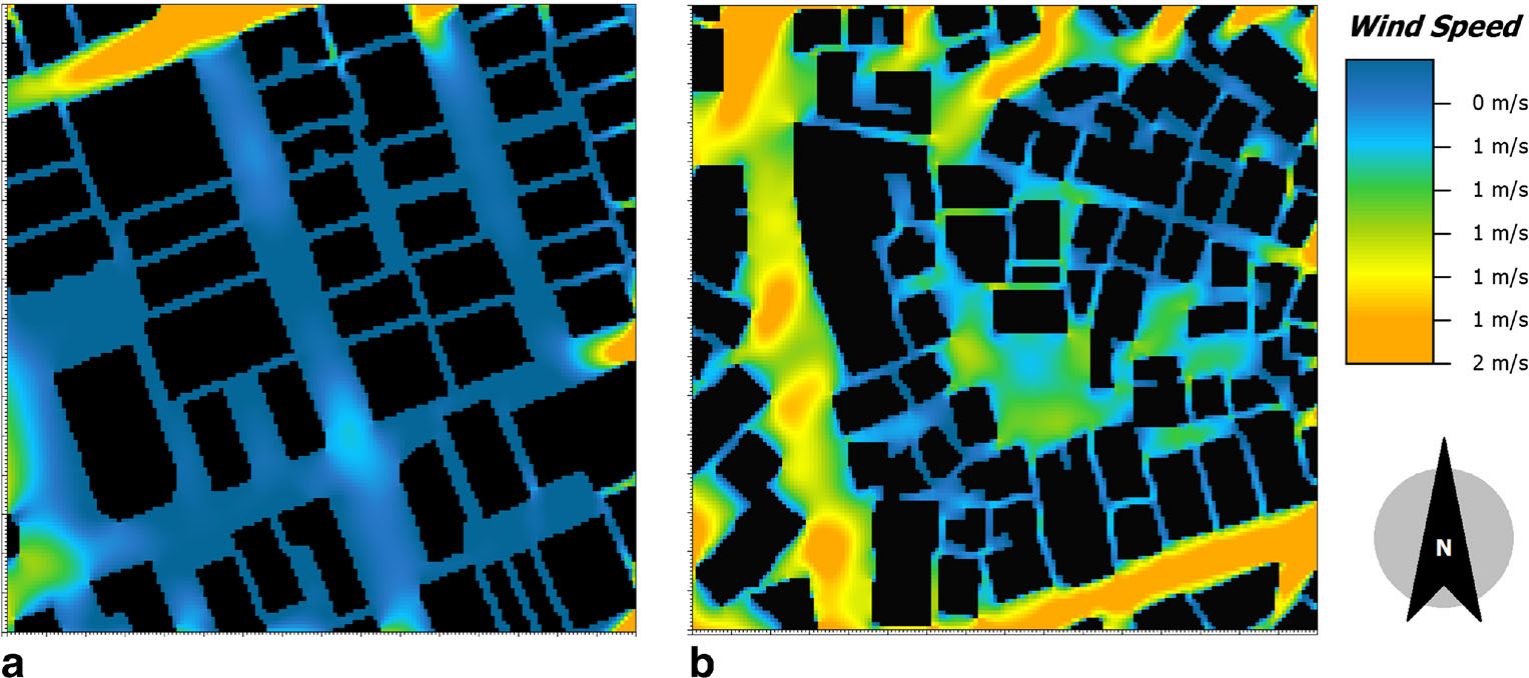
Spatial variations of the wind speed with a direction of 45° in February at 14:00 in **a** Kariakoo and **b** Manzese at pedestrian height (2 m)

Due to the differences in building heights, spaces between buildings and street orientation, the average *W*_s_ varied significantly from one area to another (Table 2). *W*_s_ is highest in Manzese (0.7 ms^−1^) and lowest in Kariakoo (0.3 ms^−1^), which is due to higher buildings and more narrow spaces between buildings in Kariakoo. The spatial variations of the wind speed in Kariakoo and Manzese with a wind direction 45° in February at 14:00 are shown in Fig. 5.

### Spatial and temporal variation of thermal comfort

**Fig. 6:**
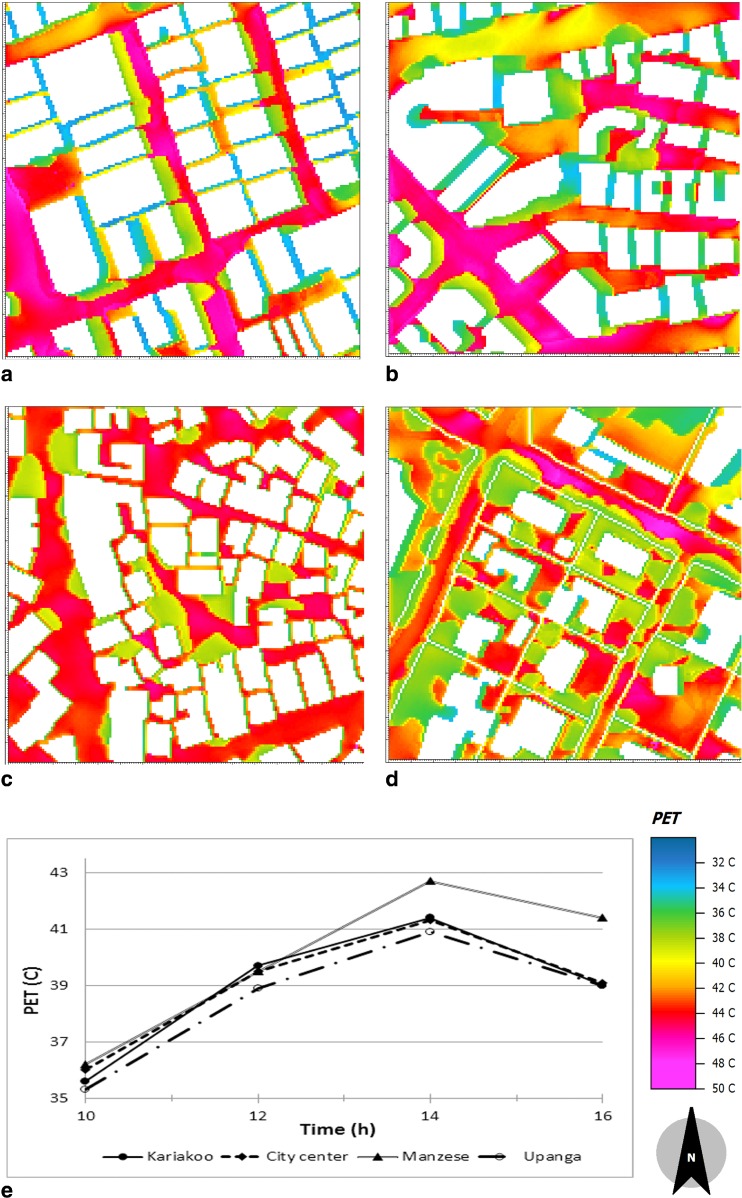
Spatial variation of the physiological equivalent temperature (PET) at pedestrian height (2 m) for the areas simulated in February at 14:00 where **a** is Kariakoo, **b** is City Center, **c** is Manzese, and **d** is Upanga. **e** is the temporal average physiological equivalent temperature (PET) for the areas simulated, i.e., City Center, Kariakoo, Manzese, and Upanga

Figure 6 illustrates the spatial and temporal variations of PET at 2 m in the four areas. As shown, the spatial distribution of PET is similar to that of MRT (Fig. 3), which implies that radiant heating constitutes a large part of the heat load (heat stress). It is clear that the main wide streets are the most stressful spots (PET varies from 40 to 47 °C). Similar to MRT, the spaces between buildings in Kariakoo and the City Center are less stressful than the wide streets. On the other hand, due to the low building heights in Manzese and Upanga, the spaces between buildings are more stressful compared to Kariakoo and the City Center. The spaces between buildings in Upanga are the least stressful of the areas due to the vegetation (the improvement of PET is about 7–11 °C). Similar results were found by Johansson and Yahia (2012) who pointed out that the thermal conditions in warm-humid Guayaquil, Ecuador could be improved by about 10 °C PET by providing shading through trees or shading devices.

Figure 6e shows the area-averaged temporal variation of PET for the studied areas. The results show the same tendency of PET variation during the day in the four areas. However, the area Manzese is the most stressful at 14:00 (the average PET is 42.2 °C) and the ∆PET between 14:00 and 16:00 is 1.3 °C. Due to the vegetation cover in Upanga, the average PET values at the all studied hours are slightly lower than in the other areas, and the ∆PET between 14:00 and 16:00 is 2.3 °C. This agrees well with other studies such as Alexandri and Jones (2008) and Johansson et al. (2013).

### Seasonal variation of thermal comfort and effect of vegetation

**Fig. 7:**
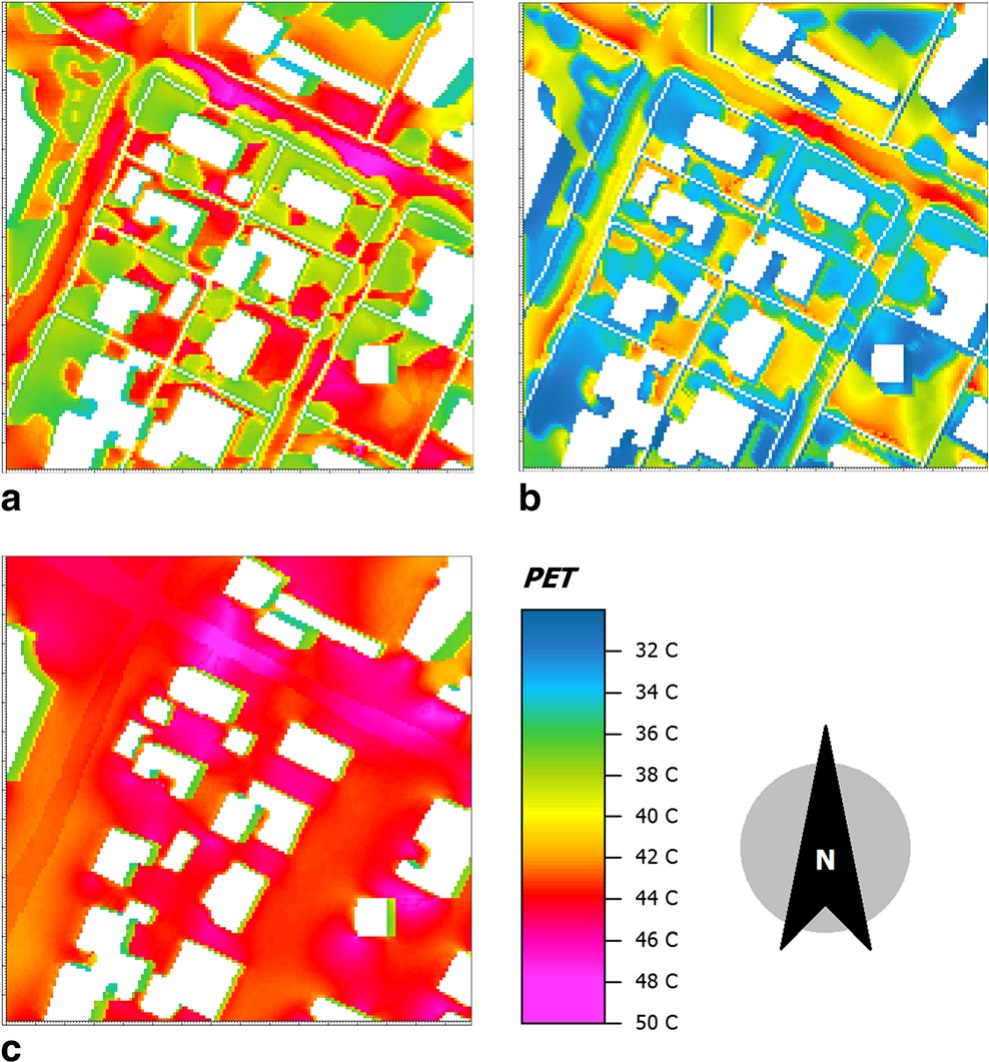
Spatial variation of the physiological equivalent temperature (PET) at pedestrian height (2 m) in Upanga (with vegetation) at 14:00 in **a** February and **b** July whereas **c** is the simulated spatial PET variation in Upanga in February at 14:00 without vegetation

The seasonal variation of thermal comfort and the effect of vegetation are shown for the area Upanga. The comparison of PET at 2 m between the months February (warm season) and July (cool season) at 14:00 is shown in Fig. 7a, b. The results for February—which is one of the warmest months—show that the PET in Upanga varies between 33 and 48 °C (with an average value in the whole area of about 41 °C) whereas in July—which is one of the coolest months—the PET varies between 31 and 44 °C (average about 36 °C). The results show that the ∆PET between February and July is 4 °C (as an average value). The cool season is thus considerably more comfortable than the warm season. Nevertheless, at some spots (especially at the middle of the streets) PET reaches as high as 44 °C in July (Fig. 7b).

The effect of vegetation on thermal conditions in Upanga (with and without vegetation) is shown in Fig. 7a, c and in Table 3. When about one third of the total area is planted, *W*_s_ is reduced by more than 50% and MRT by 7 °C. The average PET in the area is reduced from 44 to 40 °C. The improvement in thermal comfort mainly occurs at the spots where trees are added (the reduction under the trees may reach 14 °C PET). Although the cooling effect by shading is to some extent counterbalanced by the reduction of wind speed, vegetation at street level in warm humid climates considerably enhances the thermal environment outdoors (Johansson and Yahia 2015).

**Table 3:** Effect of green area coverage on wind speed, MRT, and thermal comfort at pedestrian height (2 m) in Upanga (with and without vegetation) in February at 14:00

	Green area coverage (%)	Ws (ms^−1^)	MRT (°C)	PET (°C)
Upanga with vegetation	33.8	0.59	50	40
Upanga without vegetation	0	1.3	57	44

### Sensitivity analysis of wind and shade on thermal comfort

**Fig. 8:**
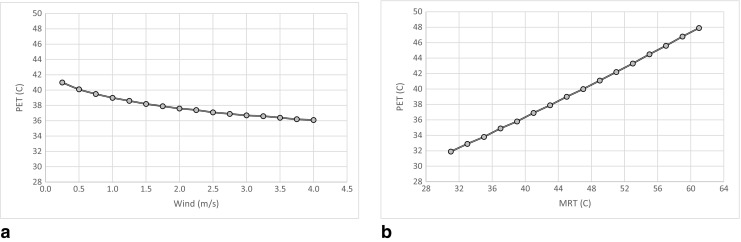
Effect of **a** wind speed and **b** mean radiant temperature (MRT) on physiological equivalent temperature (PET) in Kariakoo at 14:00

In order to analyze the cooling effect by wind and shade on outdoor thermal comfort conditions, a sensitivity analysis was carried out. Figure 8 shows the simulated relationship between *W*_s_ and MRT on PET in Kariakoo at 14:00. The range of *W*_s_ and MRT in Fig. 8 represents the values found in the simulations whereas the values of *T*_a_ (31.9 °C) and RH (65.5%) were kept constant. The investigation shows that increasing the wind speed from 0.2 to 4 ms^−1^ has a positive effect by reducing PET by about 5 °C (Fig. 8a). On the other hand, decreasing MRT from 61 to 31 °C has a more decisive effect on PET (16 °C reduction, Fig. 8b). This result shows that the PET is more sensitive to MRT than to *W*_s_, and therefore decreasing MRT through shade will have a bigger effect on PET than increasing W_s_ in Dar es Salaam.

### The effect of SVF, built area coverage and FAR on PET

SVF is defined as the portion of the sky which can be seen from a point on a surface. The built area coverage is the amount of the area covered by buildings, whereas FAR is defined as the total floor area of a buildings divided by the total area of the plots.

**Fig. 9:**
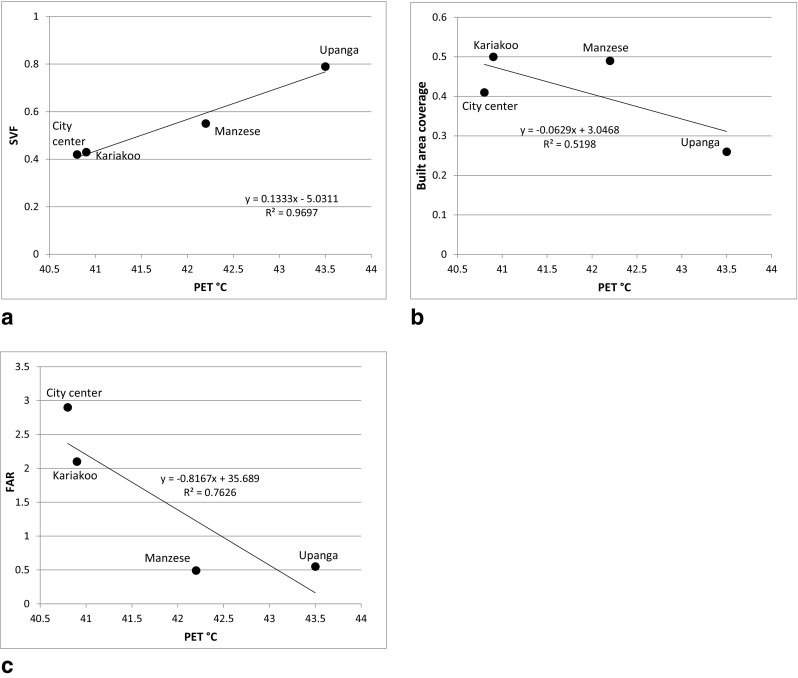
Effect of sky view factor (SVF), built area coverage and floor area ratio (FAR) on physiological equivalent temperature (PET)

Figure 9a shows a strong linear relationship (*R* ^2^ = 0.97) between the area-averaged SVF calculated by ENVI-met and average PET at 2 m. This agrees well with Chen et al. (2012) who found a linear relationship between SVF and *T*_a_ in warm-humid Hong Kong. This means that the more open the urban morphology to the sky, the more stressful thermal conditions occur. The results reveal that the minimum SVF is 0.4 at the City Center and this corresponds to 40.8 °C PET. The maximum SVF is 0.8 at Upanga (without vegetation) and it corresponds 43.5 °C PET (Fig. 9a). This means that an increase of 3 °C PET occurs when SVF increases from 0.4 to 0.8. This can be explained by the fact that the compact urban morphologies reduce the time of solar exposure and also reduce the amount of direct solar radiation which reaches the ground surface. This agrees with Mills (1997) who reported that the solar exposure and SVF are two key factors which determine the daily heat balance in the urban structure.

Contrary to SVF, lower built area coverage and lower FAR lead to higher PET (Fig. 9b, c). Built area coverage has the weakest linear relationship (*R* ^2^ = 0.52). The reason for this is that the built area coverage does not include the effect of building height. FAR, which includes the building height, has a stronger correlation than built area coverage (*R* ^2^ = 0.76) but weaker than SVF (*R* ^2^ = 0.97). One reason is that FAR only includes the effect of buildings and not vegetation. The results thus imply that SVF is a better indicator of thermal comfort than the built area coverage and FAR.

## Discussion

### Physical and thermal characteristics of the studied areas

This study reveals that shade is crucial to maintain comfortable thermal conditions in cities located in a warm climate. The area Manzese, which consists of mainly one-story buildings, for example receives much more solar radiation at the ground than the other studied areas, which consist of more high-rise buildings (Fig. 3). Manzese has a lower frequency of low MRTs (i.e., shade) and a higher frequency of high MRTs than the other areas. The results are in line with previous studies, e.g., Emmanuel et al. (2007) and Yahia (2014).

Closely spaced high-rise buildings have, however, a negative impact on ventilation. The average wind speed in the dense high-rise area of Kariakoo is for example less than half of that of the low-rise Manzese, see Table 2. However, when comparing the maximum PET of these two areas, Manzese is more uncomfortable and the shade seems to be a more important factor than wind speed. Therefore, decreasing solar radiation through shade will have a bigger effect on thermal comfort than promoting the wind speed in the warm-humid Dar es Salaam. Although MRT is one of the most important meteorological parameters that controls the human energy balance and human biometeorology (Mayer and Höppe 1987), the role of wind speed should not be neglected in warm-humid climates. Therefore, the combination of enhanced wind speed and low MRT to increase the level of comfort outdoors is needed and will be the subject of the future studies.

This study shows a linear relationship between SVF, built area coverage and FAR and thermal comfort expressed as PET. However, it is illustrated that the SVF (including both buildings and vegetation) and FAR better explain the variation in PET than built area coverage (Fig. 9). This is mainly because the SVF and FAR consider the dimension of height and not only two dimensions. In the hot humid climate of Hong Kong, Chen and Ng (2011) argued that SVF is the most suitable indicator to describe the building density in relating with air temperature variation for complex urban environment. He et al. (2015) concluded that the areal mean SVF within a specific radius, which uses a high-resolution raster digital elevation model that consists of both buildings and ground height, is the most suitable and effective in representing and describing urban scale spatial variations of urban geometry that correspond to the master plan of a given city.

Regarding the thermal conditions, this study found the area-averaged maximum PET values (at 14:00) in the warm season to be above 40 °C in all areas studied. This agrees well with the study in Dar es Salaam by Ndetto and Matzarakis (2015) who argued that in the afternoon within the period November to April (and especially in February) extreme heat stress occur (PET above 41 °C). Indeed, during the warm season PET values are clearly above the comfort range for Dar es Salaam of 23–31 °C PET recently suggested by Ndetto and Matzarakis (2016). Thus, although the local population accept rather high temperatures, the afternoon thermal conditions during the warm season are likely to be perceived as stressful. Only in the narrow spaces between tall buildings in Kariakoo and in the City Centre PET values are close to the upper limit of comfort. For the cool season, this study showed that the area-averaged maximum PET values are about 36 °C. However, some spots in the spatial PET distribution map for Upanga (Fig. 7b), mainly under trees, would be defined as “comfortable” according to Ndetto and Matzarakis (2016).

### Landscaping, ventilation, and shading strategies in Dar es Salaam

To use vegetation is obviously a useful strategy for creating shade in outdoor urban spaces in Dar es Salaam, especially if buildings are low-rise (Figs. 6 and 7). Due to the large amount of vegetation in Upanga, the average PET values at all studied hours (10:00, 12:00, 14:00, and 16:00) are slightly lower than in the other areas (Fig. 6e). The effect of green area coverage on thermal comfort agrees well with other studies such as Alexandri and Jones (2008) and Johansson et al. (2013). According to Fahmy et al. (2010), who investigated the minimum LAI value of a tree needed to produce maximum shadow in hot dry Cairo, a tree crown LAI of 1 intercepted about half of the short wave direct radiation whereas a LAI of 4 it intercepted basically 100% of the radiation. In this study, dense trees (LAI = 4.73), very dense trees (LAI = 9.35), and grass (LAI = 0.03) contributed to a reduction of MRT by 7 °C and PET by 4 °C (Table 3). It should be noted that the effect of vegetation on thermal comfort will be even bigger during clear sky conditions. Although grass gives a low surface temperature, Shashua-Bar et al. (2011) concluded that using grass brings little improvement to the thermal environment compared to using trees since the latter also limit the amount of both direct and reflected solar radiation.

In warm humid climates, it is always recommended to provide adequate air ventilation of urban districts. However, blockage to air movements at the pedestrian level by additional planting should be avoided which requires a suitable form of the trees. Ng (2009) argues that it is needed to plant tall trees with wide and dense canopies along streets, plaza entrances and setback areas for maximizing pedestrian comfort. More studies are needed to investigate the most efficient type of trees that can provide shade without reducing ventilation in the city of Dar es Salaam. This has to be based on the type of trees available in the region, and the assessment of their LAI values in different seasons.

### Recommendations to enhance shade

**Fig. 10:**
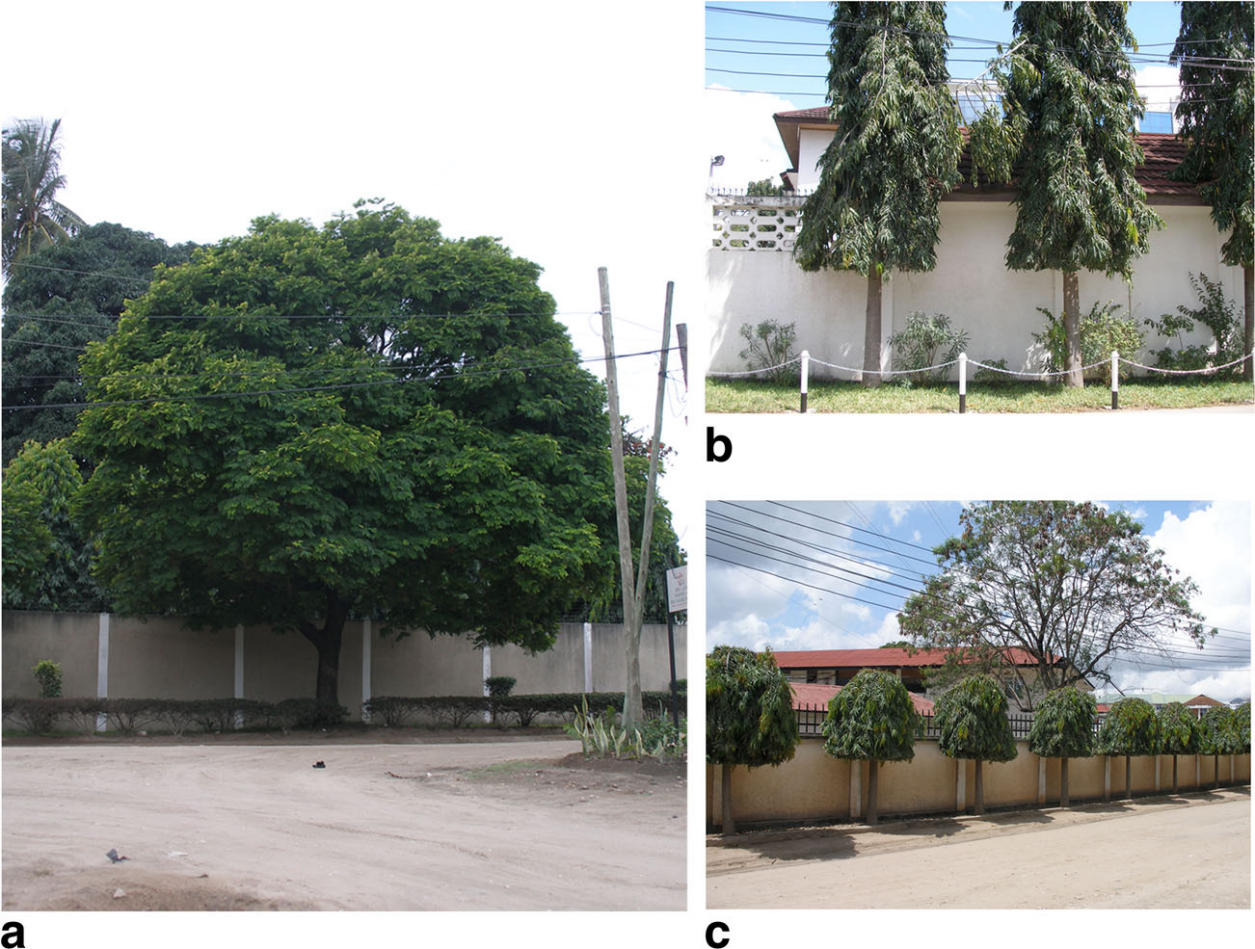
Example of typical trees used in Dar es Salaam

**Fig. 11:**
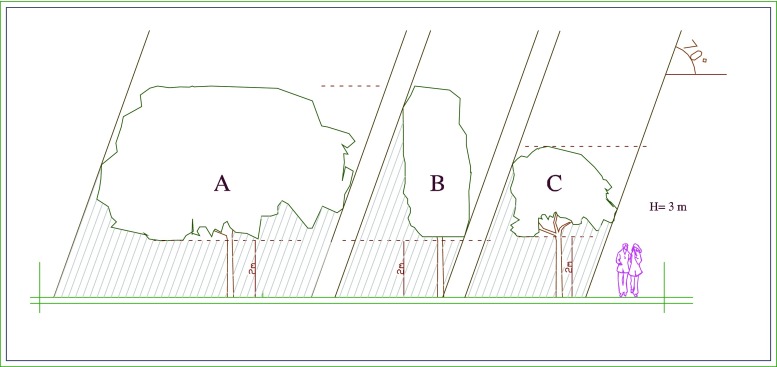
Amount of shade created by typical trees used in Dar es Salaam when the sun angle is about 70° (February at 14:00). The letters *A*, *B*, and *C* refer to the trees shown in Fig. 9

**Fig. 12:**
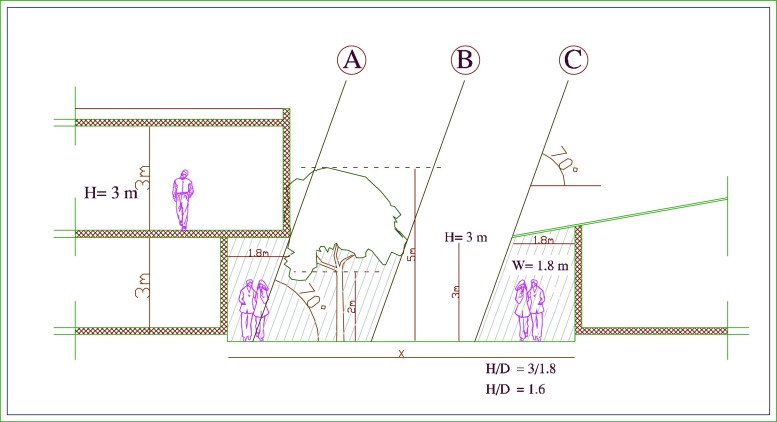
Proposed architectural elements in Manzese where *A* arcades, *B* trees, and *C* roof extensions

This study recommends having a combination between architectural elements and vegetation to improve the thermal environment for humans. Some examples are shown in Figs. 10, 11, and 12. In Dar es Salaam, there are many different types of street trees which provide different amounts of shade. Figures 10 and 11 show three types of trees, A which is a very dense tree with large crown, B which has a cylindrical shape, and C which is a dense tree with a spherical crown. Whereas shapes B and C give limited shade, large, and dense tree crowns such as A can be used to create continuous shade if trees are planted sufficiently close. To encourage wind flow and activities under the tree crowns, it is recommended to have no branches and leaves up to at least 2 m height.

The results of this study clearly revealed that the area which most lack shading is the informal settlement Manzese. To improve the thermal situation in such low-rise neighborhoods, architecture elements such as arcades (see detail A in Fig. 12) and roof extensions (detail C) can be considered. Adding trees as landscape elements will help to provide additional shade (detail B), see also Yahia and Johansson 2014. Similar measures will of course improve the situation in other neighborhoods as well.

## Conclusions

This study investigated the relationship between urban design, urban microclimate, and outdoor comfort in four different built-up areas, with different urban morphologies, in the city of Dar es Salaam, during the warm and cool seasons.

Both during the warm and cool seasons, the thermal conditions in the afternoon are stressful; however, the average PET values of the areas are about 4 °C lower in the cool season. The thermal condition vary greatly within the areas and in shaded places such as in narrow streets with high buildings and under dense trees PET values may reach comfortable levels even in the afternoon, especially during the cool season.

The study illustrated that the areas with low-rise buildings lead to more stressful urban spaces than the areas with high-rise buildings. The study showed that the compact urban morphologies reduced the time of solar exposure and also reduced the amount of direct solar radiation which reached the ground surface. It is also observed that the sky view factor (SVF) better explained the variation in PET than the built area coverage and the floor area ratio (FAR). It is also shown that the use of green cover and especially dense trees helps to enhance the thermal environment. The reduction of PET under trees may reach 14 °C.

This study revealed that the PET is more sensitive to changes of the mean radiant temperature than changes of wind speed.

It was also concluded that further studies are needed to investigate the relationship between wind patterns, urban morphology, urban design elements, and landscaping.
